# Frequency and associated risk factors of non-fatal overdose reported by pregnant women with opioid use disorder

**DOI:** 10.1186/s13722-018-0126-0

**Published:** 2018-12-14

**Authors:** Sarah M. Bagley, Howard Cabral, Kelley Saia, Alyssa Brown, Christine Lloyd-Travaglini, Alexander Y. Walley, Ruth Rose-Jacobs

**Affiliations:** 1 Department of Medicine, Boston University School of Medicine, Boston Medical Center, Boston, MA 02118 USA; 2 Department of Pediatrics, Boston University School of Medicine, Boston Medical Center, Boston, MA 02118 USA; 3 School of Public Health, Boston University, Boston, USA; 4 Department of Obstetrics and Gynecology, Boston University School of Medicine, Boston Medical Center, Boston, USA; 5 Northeastern University School of Public Health, Boston, USA

**Keywords:** Opioids, Overdose, Pregnancy, Naloxone

## Abstract

**Background:**

Little is known about opioid overdose or naloxone access among pregnant women.

**Objectives:**

The objectives of this study were to determine the prevalence of non-fatal overdose, risk factors for overdose, and naloxone access among third trimester women in treatment for opioid use disorder.

**Methods:**

We collected baseline data from a case management parental-support intervention study. To explore the association of variables with past year overdose, we used Wilcoxon rank-sum test, Chi square or Fisher’s exact tests.

**Results:**

Among 99 participants, 14% (95% CI 7–21%) reported past year overdose and 67% (95% CI 57–76%) had received overdose education and a naloxone kit. Younger age was the only variable associated with past year overdose.

**Conclusions:**

In this sample, past year non-fatal overdose was common, younger age was a risk factor, and most participants had received a naloxone kit. Further work is needed to understand whether younger age is a risk factor in the general population of pregnant women with opioid use disorder and to identify other potential risk factors for overdose in this population.

## Introduction

Opioid use disorder among pregnant women quadrupled from 1999 to 2014 [5] and between 2004 and 2013, the incidence of neonatal intensive care unit admissions for neonatal abstinence syndrome increased from 7 cases to 27 cases per 1000 admissions per year [8]. This evidence of increased burden of the opioid epidemic on pregnant women has paralleled an increase in opioid-related overdose deaths among women that has been faster than among men [3, 6]. Recent research in Massachusetts demonstrated that 2.3% of newborns were delivered to women with opioid use disorder and detailed the falling and rising risk of opioid overdose across pregnancy and post-partum periods [9]. Little is known about risk factors for overdose during pregnancy. Naloxone access, however, is a key strategy in addressing rising opioid-related overdose deaths for all populations in the United States [2]. The objectives of this study were to determine the prevalence of reported non-fatal overdose, identify risk factors for overdose, and describe naloxone access in a sample of third trimester pregnant women in treatment for opioid use disorder.

## Methods

We collected baseline data from a randomized controlled trial of a case management parental support intervention among pregnant women with opioid use disorders. Participants were recruited at an urban, academic obstetrics clinic specializing in pregnant women with substance use disorder. Maternal inclusion criteria were: treatment with methadone or buprenorphine, prenatal care and intent to deliver at our academic center, due date within 3 months of enrollment, ≥ 18 years, singleton pregnancy, not incarcerated, ability to give consent, and intent to stay in area after delivery. Women planning to relinquish custody after delivery were excluded. Only women who met inclusion criteria for the intervention trial had baseline information collected.

The questionnaire, administered in the third trimester, prior to randomization, included demographics, major mental health diagnoses (depression, panic attacks/anxiety disorder, bipolar disorder, schizophrenia, post-traumatic stress disorder), opioid overdose history and naloxone access. Overdose history was determined by asking, “Have you ever overdosed?” and if positive: “In the past year, how many times have you overdosed? Naloxone access was determined by asking: “Have you ever received an overdose rescue kit that includes Narcan to use JUST IN CASE of an overdose?” and “Where did you receive the overdose rescue kit?”

The study was approved by the Boston University Medical Center Institutional Review Board and is registered at clinicaltrials.gov NCT02334111.

Using SAS 9.3 to explore the association of variables with past year overdose and no past year overdose, we conducted a Wilcoxon rank-sum test for continuous variables and Chi square or Fisher’s exact tests for categorical variables. We combined the “no past year overdose” and “never overdosed” categories to one “no past year overdose” category because those participants would not have had an overdose during pregnancy.

## Results

One hundred participants were recruited to the intervention study, however, one had missing overdose data and was not included in the analysis. The mean age was 28.5 years, 83% had at least a high school education, 73% were White/non-Hispanic, 82% were married or in a relationship, 74% were unemployed and 86% reported a major mental health diagnosis.

Among participants, 14% reported past year overdose; 30% reported lifetime overdose history (but no past year overdose), and 56% reported no overdose history. In the comparisons of past year overdose to no past year overdose, the only significant association with past year overdose was younger maternal age (p = 0.0081) (Table 1). Notably, of the 14 women who reported a past year overdose, all reported a major mental illness diagnosis. Of those with no past year overdose, 84% (n = 71) reported a major mental illness diagnosis. Among those who overdosed in the past year, the median number of reported overdoses during that time was one, and the maximum number was seven.

**Table 1:** Baseline characteristics of pregnant women with opioid use disorders stratified by past year overdose history

Variable	Total N = 99 (95% CI)	Overdose within last year n = 14 (95% CI)	No past year overdose n = 85 (95% CI)	*p* Value
Mean maternal age	28.5 (27.5–29.5)	25.2 (23.0–27.4)	29.0 (28.0–30.1)	0.0081*
White/non-hispanic	72 (72.7%) (64.0–81.5%)	13 (92.9%) (79.4–100.0%)	59 (69.4%) (59.6–79.2%)	0.1038
Married/in a relationship	81 (81.8%) (74.2%, 89.4%)	11 (78.6%) (57.1%, 100.0%)	70 (82.4%) (74.3%, 90.5%)	0.7152
Public insurance	92 (92.9%) (87.9%, 98.0%)	12 (85.7%) (67.4%, 100.0%)	80 (94.1%) (89.1%, 99.1%)	0.2572
Age at opioid initiation	20.2 (19.2–21.1)	18.3 (17.2–19.4)	20.5 (19.4–21.6)	0.1227
Any history of mental health diagnosis	85 (85.9%) (79.0–92.7%)	14 (100.0%) (100.0–100.0%)	71 (83.5%) (75.6–91.4%)	0.2084
Number of mental health diagnoses	2.4 (2.1–2.7)	3.0 (2.2–3.8)	2.3 (2.0–2.6)	0.1049
2 or More mental health diagnoses	72 (72.7%) (64.0–81.5%)	12 (85.7%) (67.4–100.0%)	60 (70.6%) (60.9–80.3%)	0.3389
Ever received a naloxone kit	66 (66.7%) (57.4–76.0%)	11 (78.6%) (57.1–100.0%)	55 (64.7%) (54.6–74.9%)	0.3742

*Significant result

Sixty-seven percent (95% CI 57–76%) of participants had ever received a naloxone rescue kit (Table 1). Naloxone sources included inpatient detoxification programs (n = 28), needle exchanges (n = 16), friends/acquaintances (n = 10), methadone programs (n = 10), emergency departments (n = 4), and drop-in centers (n = 4).

## Discussion

About 1 in 7 pregnant women treated for an opioid use disorder reported a past year overdose. Younger age was associated with greater risk of past year overdose compared to no past year overdose. As far as we know, finding of younger age associated with past overdose in pregnant women has not previously been documented in the literature. This study found that 100% (n = 14) of women with an overdose in the past year and 84% (n = 71) of women with no past year overdose self-reported a major mental health diagnosis. This is consistent with other research findings that substance use may be used to relieve the symptoms associated with mental health disorders [7].

The majority of the sample had previously received a naloxone rescue kit, reflecting increased community access, although with the continued increase in opioid-related deaths there is still room for improvement. In Massachusetts, where this study was conducted, overdose education and naloxone (OEND) access was common at many addiction treatment, harm reduction, and healthcare venues, but was not a specific component of the intervention offered in this study [12]. OEND has been incorporated into settings where recruitment for this study occurred, such as medication treatment programs.

Prior work has shown that detoxification centers, prisons and other abstinence-based treatment programs present an excellent opportunity to train individuals on use and distribute naloxone rescue kits before release [1, 11]. Pregnancy provides another opportunity to target this high-risk population. Pregnant women, motivated by their pregnancy, are engaged in the health care system and may be more receptive to education and prevention messages [4, 10]. In addition, although it is common to focus on recovery during pregnancy, relapses and overdoses can occur. This study highlights that pregnant women are another group at risk for overdose and should be offered and have access to OEND. Since this population is most likely receiving prenatal care, obstetricians and other providers caring for this population should incorporate OEND into their services.

Study limitations included small sample size, and a reliance on self-reported data. The dates of overdose events were not collected. Because the window we asked about for overdose was 12 months and all the participants were in the third trimester of their pregnancies, we do not know if overdose occurred during or before pregnancy, and whether overdose might have been a factor in the decision to seek substance use disorder treatment. In addition, because baseline information was collected during the third trimester of pregnancy among women intending to retain custody, participants may have under-reported their overdose history.

Our findings support targeted overdose prevention efforts among pregnant women with opioid use disorder that include naloxone rescue kits. Further research in this high-risk population, including understanding why younger maternal age may be a particular risk factor, is warranted.
